# Abundant and species-specific *DINE-1 *transposable elements in 12 *Drosophila *genomes

**DOI:** 10.1186/gb-2008-9-2-r39

**Published:** 2008-02-21

**Authors:** Hsiao-Pei Yang, Daniel A Barbash

**Affiliations:** 1 Institute of Genetics, National Yang-Ming University, Taipei 112, Taiwan; 2 Department of Molecular Biology and Genetics, Cornell University, Ithaca, NY 14853, USA

## Abstract

**Background:**

Miniature inverted-repeat transposable elements (MITEs) are non-autonomous DNA-mediated transposable elements (TEs) derived from autonomous TEs. Unlike in many plants or animals, MITEs and other types of DNA-mediated TEs were previously thought to be either rare or absent in *Drosophila*. Most other TE families in *Drosophila *exist at low or intermediate copy number (around < 100 per genome).

**Results:**

We present evidence here that the dispersed repeat *Drosophila *interspersed element 1 (*DINE-1*; also named *INE-1 *and *DNAREP1*) is a highly abundant DNA-mediated TE containing inverted repeats found in all 12 sequenced *Drosophila *genomes. All *DINE-1*s share a similar sequence structure, but are more homogeneous within species than they are among species. The inferred phylogenetic relationship of the *DINE-1 *consensus sequence from each species is generally consistent with the known species phylogeny, suggesting vertical transmission as the major mechanism for *DINE-1 *propagation. Exceptions observed in *D. willistoni *and *D. ananassae *could be due to either horizontal transfer or reactivation of ancestral copies. Our analysis of pairwise percentage identity of *DINE-1 *copies within species suggests that the transpositional activity of *DINE-1 *is extremely dynamic, with some lineages showing evidence for recent transpositional bursts and other lineages appearing to have silenced their *DINE-1*s for long periods of time. We also find that all species have many *DINE-1 *insertions in introns and adjacent to protein-coding genes. Finally, we discuss our results in light of a recent proposal that *DINE-1*s belong to the *Helitron *family of TEs.

**Conclusion:**

We find that all 12 *Drosophila *species with whole-genome sequence contain the high copy element *DINE-1*. Although all *DINE-1*s share a similar structure, species-specific variation in the distribution of average pairwise divergence suggests that *DINE-1 *has gone through multiple independent cycles of activation and suppression. *DINE-1 *also has had a significant impact on gene structure evolution.

## Abstract 2

Evidence is presented that DINE-1 is a highly abundant miniature inverted-repeat transposable element (MITE) family present in all 12 Drosophila species with whole-genome sequence available.

## Background

Interspersed repetitive sequences are ubiquitous to all eukaryotic organisms, and make up a significant portion of the genome [1-7]. These sequences are mostly transposable elements (TEs) or TE-derived sequences, and they play important roles in the evolution of chromosome organization and genome complexity [8].

Based on their mechanism of transposition, TEs can be divided into two classes: class I comprises retrotransposons, which transpose through RNA-mediated mechanisms, and class II comprises transposons, which mobilize through DNA-mediated mechanisms [9,10]. Depending on their ability to direct their own transposition, each class of TEs can contain two types: autonomous and non-autonomous copies. Autonomous TEs code for the proteins that are required for their transposition, and are mobilized in *cis*. Non-autonomous TEs are mobilized in *trans *by enzymes produced from autonomous elements. Well-known examples include the vertebrate retroelements LINEs (long interspersed elements) and SINEs (short interspersed elements). The mobilization of non-autonomous SINEs requires retrotransposase from autonomous LINEs, and these elements co-evolve in a highly species-specific manner [11,12]. Another example is the miniature inverted-repeat transposable elements (MITEs) found in many plant genomes. MITEs are non-autonomous DNA elements (class II) that originated from a subset of autonomous DNA transposons [13]. They are characterized by short sequences with no coding capacity, flanked by terminal (or occasionally subterminal) inverted repeats (TIRs) and very short direct repeats caused by target site duplication (TSD). MITEs have no internal homology to their parental autonomous transposons and often include non-homologous sequences in their internal regions. MITEs have also been found in several animal genomes, including *Caenorhabditis elegans*, mosquitoes, fish and humans (reviewed in [14]). Both SINEs and MITEs are highly abundant (usually > 1,000 copies per genome) in many host species across a broad taxonomic range. Because of their high abundance and active movement, and their frequent association with genes [15,16], MITEs have had a significant impact on the evolution and complexity of eukaryotic genomes.

TE activity and evolution have been intensively studied in *Drosophila *and many families of TEs have been described [5,6,17-19]. Most TEs are at low or intermediate copy number in *D. melanogaster*. MITEs and SINEs have been previously reported as being either rare or absent in most species of this genus that have been examined [20]. *D. melanogaster DINE-1 *(*Drosophila *interspersed element 1; also named *INE-1*, *DNAREP1*) is an exception to these observations [21]. *D. melanogaster *contains thousands of copies of *DINE-1 *[19]. All copies appear to be non-autonomous, and analyses of their divergence patterns suggest that *D. melanogaster DINE-1 *has been inactive for over 4 million years [22]. Although *DINE-1 *was originally suggested to be a SINE-like retroelement, we have suggested that it is more likely to be a MITE, based on analysis of *DINE-1 *elements in *D. yakuba *that show evidence of recent transpositional activity [23]. We discuss below the structural features of *DINE-1 *supporting this designation, as well as the more recent proposal [24] that *DINE-1*s are members of the *Helitron *family of TEs.

Several earlier studies found high copy TEs that we here classify as *DINE-1*. Vivas *et al*. [25] discovered an element in *D. subobscura *called *GEM *that is composed of repetitive modules, one of which they also found in the *D. melanogaster *and *D. virilis *genomes. Miller *et al*. [26] characterized an abundant element called *SGM *in *D. subobscura*, *D. guanche *and *D. madeirensis*, noted its similarity to *GEM*, and also described that other species, including *D. melanogaster *and *D. virilis*, have similar sequences; *GEM *and *SGM *are the same as *DINE-1*. Wilder and Hollocher [27] subsequently discovered an element in a number of *Drosophila *species that they called *mini-me *and noted its similarity to *D. melanogaster DINE-1*. However, a comprehensive assessment of the abundance and transpositional dynamics of *DINE-1 *has not been reported. Here we expand our study of the evolutionary dynamics of *DINE-1 *using the recently available genome sequences of 12 *Drosophila *species [7]. We found that *DINE-1*-related sequences are not only highly abundant in all 12 species, but also share a similar sequence structure, suggesting that a common mechanism was used for their transposition. Different lineages, however, show different distributions of divergence, suggesting that *DINE-1 *has gone through multiple cycles of transposition and subsequent silencing.

## Results

### Identification and common sequence structure characteristics of *DINE-1*s in 12 *Drosophila *species

**Figure 1:**
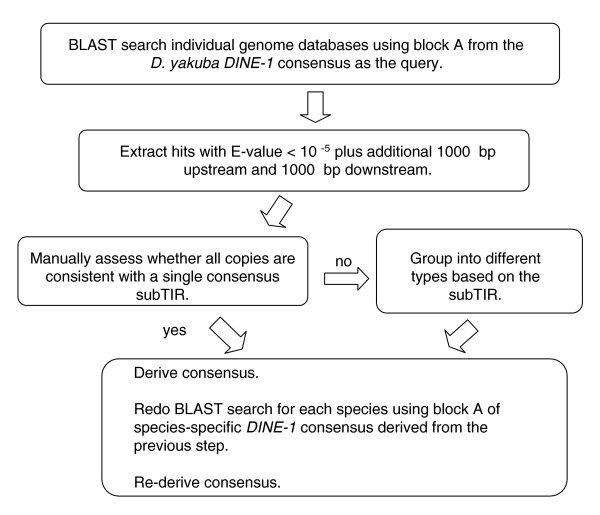
Flow chart of the strategy for identifying *DINE-1 *sequences in the 12 *Drosophila *genomes.

**Figure 2:**
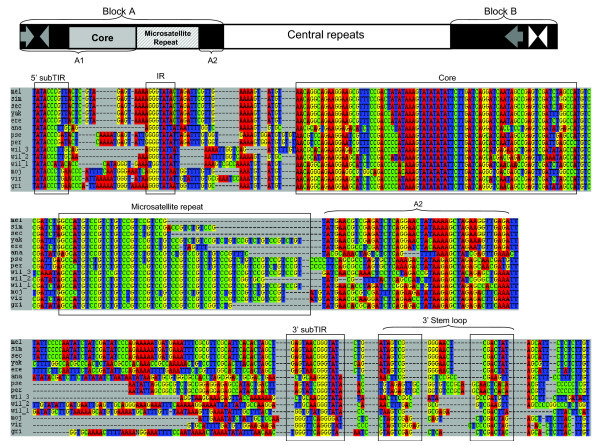
The generalized structure of *DINE-1 *sequences from 12 *Drosophila *genomes, and alignment of the *DINE-1 *consensus sequences from 12 species with each feature boxed. The element contains two conserved blocks, A and B. Within block A, the sequence can be further divided into two parts, A1 and A2, separated by a region of variable length containing the tandem repeats (CCGT)_n_(CTGT)_n_. Between blocks A and B is a region of central repeats, containing species-specific repeats. These central repeat sequences do not share homology among species; the length of the repeat unit can range from approximately 50 bp to approximately 500 bp, and the number of repeats is also variable within species. Locations of the subTIRs are shown as gray arrows; see Table 1 for precise designations of subTIR sequences. The 5' end also contains a second inverted repeat (IR) sequence that is partially complementary to the 5'-end terminal repeat and is shown as a gray arrowhead. An inverted repeat near the 3' end forms a potential stem-loop structure and is indicated by white arrowheads.

Previously, we discovered that *DINE-1 *is highly abundant and appears to have experienced a recent transpositional burst in the lineage leading to *D. yakuba *[23]. Using the *D. yakuba DINE-1 *consensus sequence, we searched using BLAST for related sequences in 11 other sequenced genomes of *Drosophila *(Figure 1). This initial screen suggested that all 12 *Drosophila *species contain hundreds to thousands of copies of *DINE-1*-related sequences. To infer the structure of *DINE-1 *in each species, we manually aligned 50 sequences with the highest BLAST scores from each species. Then we aligned together these sequences from all the species. This analysis revealed that *DINE-1*-related sequences from the ten newly analyzed *Drosophila *species share a number of structural similarities with *DINE-1 *from *D. yakuba *and *D. melanogaster *(Figure 2; Table 1; also see Additional data file 1).

**Table 1:** Abundance and sequence diversity of *DINE-1 *in 12 *Drosophila *species

Species	subTIR*	Insertion preference	No. of copies^†^	Average % identity (STD)^‡^
*D. melanogaster*	ATACCCGTTACTC	TT	355	89.66 (3.57)
*D. simulans*	ATACCCGTTACTC	TT	478	90.06 (3.36)
*D. sechellia*	ATACCCGTTACTC	TT	502	90.16 (3.38)
*D. yakuba*	ATACCCGTTACTC	TT	5,424	96.43 (3.31)
*D. erecta*	ATACCCGTTACTC	TT	1,013	91.08 (3.41)
*D. ananassae*	TATACCCTTGCAG	TT	5,027	97.15 (3.32)
*D. persimilis*	TATACCCGATACT	TT	1,103	96.63 (3.29)
*D. pseudoobscura*	TATACCCGATACT	TT	1,047	95.39 (4.24)
*D. willistoni *type 1	TATACCATACACC	TT	2,396	96.70 (4.13)
*D. willistoni *type 2	TATACCCTTGCAA	TT	2,828	96.13 (3.47)
*D. willistoni *type 3	TATACCCTTGCAG	TT	1,073	96.69 (2.93)
*D. mojavensis*	ATACCCTGAACCC	TT	5,190	93.17 (4.64)
*D. virilis*	ATACCCTGAACCC	TT	3,222	94.40 (3.20)
*D. grimshawi*	TACCCTGAACCCA	TT	334	87.89 (4.34)

*subTIR of *D. mojavensis *is from the 5' side; the 3' subTIR has a single mismatch (Figure 2). ^†^Number of hits found by BLAST search using block A consensus of *DINE-1 *of each species. Only hits > 100 bp in length were included. ^‡^Means (and standard deviations (STD) in parentheses) of percentage identity between the *DINE-1 *consensus to all BLAST hits of block A.

**Figure 3:**
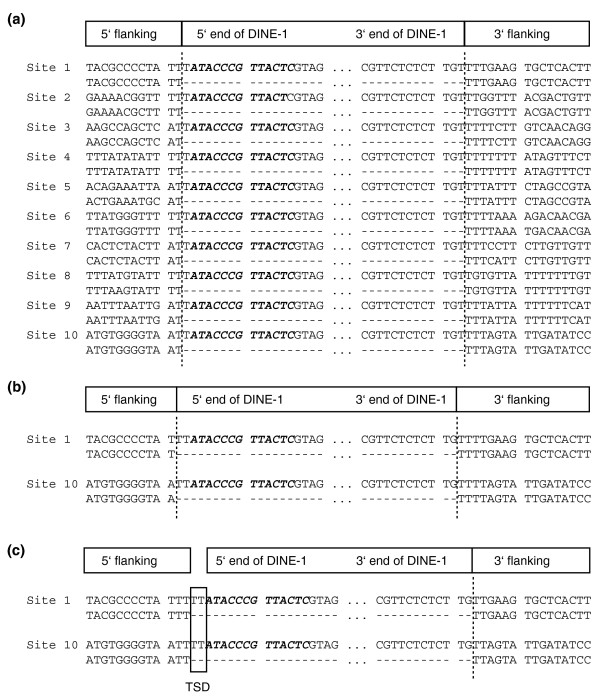
Analysis of ten sites that are polymorphic for *DINE-1 *insertions in natural populations of *D. yakuba*. For each site, the sequence from a strain containing a *DINE-1 *insertion is shown at the top, and the sequence from a strain lacking the insertion is shown at the bottom. Only the terminal sequences of *DINE-1 *and its flanking sequences are shown. The 5' subTIR is shown in bold italics. Insertions 1-3 were previously reported in [23]. The interpretation of these data depends on the designation of the *DINE-1 *termini and whether insertion causes a TSD. **(a) **Analysis using the annotation of *DINE-1 *structure presented in this paper. This annotation places the subTIR of *D. yakuba *1 bp internal to the 5' end. It also assumes that no TSD is created, in accord with the proposed mechanism of *Helitron*-type replication [24]. Under these stipulations, all ten insertions occur between the dinucleotide TT in the consensus sequence WTT (where W = A or T), and eight of ten match a longer consensus sequence of insertion after the second nucleotide in the sequence of WTTTT. **(b) **Analysis assuming the *DINE-1 *termini of [28], and *Helitron*-type replication. Only sites 1 and 10 are shown. Under this annotation, *DINE-1 *would have an insertion preference for WT dinucleotides. **(c) **Analysis assuming that the *DINE-1 *5' end begins at its inverted repeat, inserts between the dinucleotide TT and causes a 2 bp TSD, as in MITE-like DNA transposons. The TSDs caused by *DINE-1 *are boxed.

We previously defined *D. yakuba DINE-1 *as beginning at its 5' inverted repeat, based on the assumption that this sequence is a TIR [23]. We have here placed this repeat one nucleotide internal to *D. yakuba DINE-1*. This change in based on the recent designation of *DINE-1 *as a *Helitron *element [24,28]. Our designation of the boundaries of *DINE-1 *differs by one nucleotide from that in [28], based on our analysis presented below of polymorphic insertions in *D. yakuba *(Figure 3). The precise boundaries of *DINE-1 *in each species are difficult to determine because of their preference for inserting in T-rich regions. We have annotated the sequences in Figure 2 in order to maximize similarity to *D. yakuba DINE-1*. Based on this alignment the 5' inverted repeat ranges from being terminal to 2 bp internal; in all species the corresponding 3' inverted repeat is clearly internal. We thus refer to these repeats as subterminal inverted repeats (subTIRs).

The common features of *DINE-1 *from all species include: 13 bp subTIRs (the exact location of such repeats differs by 1-2 nucleotides among species); a partial inverted repeat next to the 5' subTIR; terminal regions that are relatively well-conserved within species, called blocks A and B; a GTCY-rich microsatellite repeat of variable length within block A; a variable central repeat region, which is responsible for most of the total length variation among elements; the lack of any significant open reading frames; and a propensity to insert between TT dinucleotides (discussed further below).

Our analysis revealed one novel feature not previously described for *DINE-1*, *SGM *or *mini-me*, namely a short hairpin stem-loop structure (with 7-11 nucleotide-long stems) located a few nucleotides downstream of the 3' subTIR (Figure 2). The sequence of the self-complementary stem differs among species, suggesting that compensatory mutations maintain its structure. This stem-loop may function as a terminator during rolling-circle replication (see Discussion).

Several of the features we characterized refine structural features inferred previously from *SGM *[26] and *mini-me *[27]. TIRs from *mini-me *were reported to vary from 10-20 bp in length from different species, while our analysis identified 13 bp TIRs in all species. These differences likely reflect the fact that we have analyzed many more sequences. The 13 bp subTIRs from *D. melanogaster *and *D. virilis *that we describe contain the 10 and 11 bp sequences reported by Wilder and Hollocher [27]. Likewise, the 17 bp TIR reported previously for *D. subobscura mini-me *contains the 13 bp subTIR reported here for *D. pseudoobscura*, with one internal base-pair difference. The partial inverted repeat flanking the 5' subTIR we identified is more variable than that reported based on two copies of *mini-me*, being a partial and/or interrupted repeat of the 5' subTIR in different species.

Wilder and Hollocher [27] also reported that *mini-me *elements from all species contain a highly conserved 33 bp core region. We find that this conserved core region actually extends over 90 bp, including the (TA)_4 _repeat and additional sequences 5' to this repeat. These sequences partially overlap with the LS module described for *SGM *elements [26]. The striking conservation of this core among the 12 species suggests that it is of functional significance for *DINE-1 *transposition.

We previously proposed that transposition of *D. yakuba DINE-1 *creates a dinucleotide (TT) TSD upon insertion [23]. However, based on analysis of the genome sequence from a single strain this conclusion was not definitive because some copies did not have a TT dinucleotide flanking both sides. This could be due either to accumulated mutations after insertion or because the TT site preference is not absolute. We performed a preliminary test of this hypothesis by comparing the sequences of three polymorphic insertion sites of *DINE-1 *in multiple strains of *D. yakuba *[23]. Here we extended this analysis with seven additional *DINE-1 *insertions that are polymorphic among different *D. yakuba *strains (see Materials and methods).

We found that all of these ten sites have a similar sequence structure (Figure 3). The interpretation of these data, however, depends on where precisely *DINE-1 *starts. Our previous interpretation of *DINE-1 *beginning at the 5' subTIR is consistent with insertions causing a TT TSD (Figure 3c). With the re-designation of the 5' end of *DINE-1*, these two nucleotides are instead part of the element, and *DINE-1 *would not create a TSD. The lack of a TSD is consistent with the proposal that *DINE-1*s are *Helitrons *(see below). Based on our designation of the *DINE-1 *boundaries, all *D. yakuba *insertions occur between the dinucleotide TT (Figure 3a). Using the *DINE-1 *boundaries from [28], the insertion site preference is more variable (Figure 3b). We then examined the sequences flanking the putative *DINE-1*s identified in the other *Drosophila *species. The majority (> 80%) of *DINE-1*s are flanked by TT dinucleotides. The conservation of this site preference in all 12 species, combined with the numerous other similarities described above, strongly suggests that each of these elements is in fact a species-specific *DINE-1 *and that they likely share a common mechanism of transposition.

### Relationships of *DINE-1*s within and among species

Two pieces of evidence demonstrate that *DINE-1 *is highly homogeneous within 11 of the 12 species, with *D. willistoni *discussed below as being exceptional. First, we performed BLAST searches using the 90 bp sequence of the core region, which is conserved among all types of *DINE-1*s. By comparing the TIRs and block A sequences we found that each species contains only one type of *DINE-1*. Second, we searched for *DINE-1*s in one genome using the *DINE-1 *consensus sequences from other genomes as queries, and found only the same sets of sequences.

Among the 11 species (again excluding *D. willistoni*), there are 5 different subTIRs (Table 1). All five *melanogaster *subgroup species have the same subTIR sequence. *D. ananassae *has a unique subTIR, while the closely related species *D. pseudoobscura *and *D. persimilis *share the same subTIR, as do *D. virilis *and *D. mojavensis*. The *DINE-1 *subTIR from *D. grimshawii *shares 12/13 bp with *D. virilis *and *D. mojavensis*. The central repeat is the most diverse region of *DINE-1 *among species. Even species sharing the same subTIRs, such as *D. virilis *and *D. mojavensis*, have unrelated central repeat regions.

Analysis of *D. willistoni *gave uniquely different results. *D. willistoni *contains three different subtypes of *DINE-1*s, each with different subTIRs and different central repeat sequences (Figure 2). Phylogenetic evidence presented below suggests that they have at least two independent evolutionary origins.

### Abundance and divergence of *DINE-1*s within species

*DINE-1 *is highly abundant in all 12 *Drosophila *species. It is difficult to determine an exact number because each species contains small and fragmented copies that cannot always be unambiguously identified as *DINE-1*s. We therefore used stringent criteria to identify *DINE-1*s in order to obtain a reliable comparison among species (Table 1). For example, this search identified 355 copies in *D. melanogaster *compared to previous analyses that suggested that *D. melanogaster *has approximately 1,000 copies [22]. Using identical search criteria, we found vast differences in the copy number of *DINE-1*s among species, ranging from 334 in *D. grimshawii *to 6,297 in *D. willistoni*.

We identified similar numbers of *DINE-1*s in the *D. melanogaster *sister species *D. simulans *and *D. sechellia *compared to *D. melanogaster*. In contrast, more than ten-fold more copies were identified in *D. yakuba*. This high copy number is due to the large number of closely related copies in *D. yakuba*, and is consistent with previous work that suggested that *DINE-1 *has been inactive in *D. melanogaster *but underwent a recent transpositional burst in *D. yakuba *[23].

**Figure 4:**
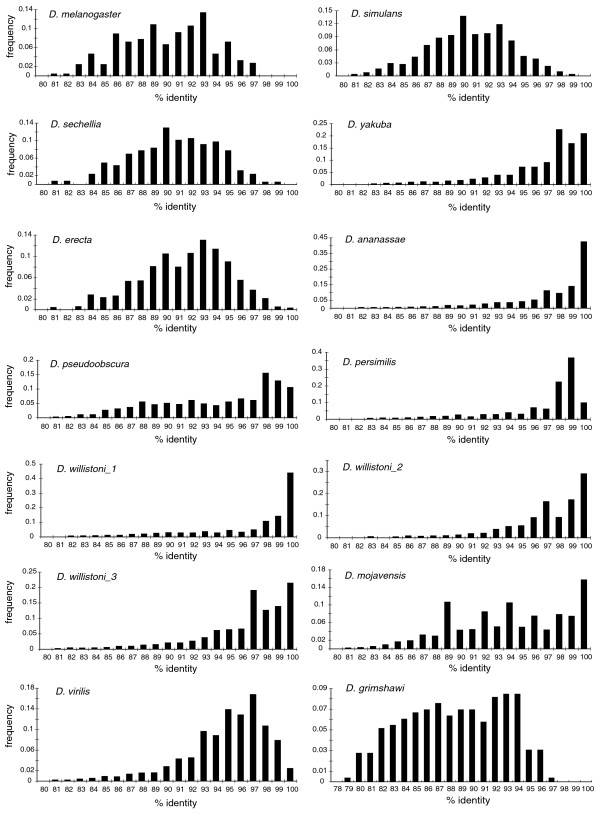
The frequency distribution of sequence identity of *DINE-1 *in different species. The percentage identity was based on BLAST search, using consensus sequences of part A1 of block A from each species as the query. To exclude short and fragmented sequences from our analysis, only hits > 100 bp were used. Note that the y-axis scale differs among species.

We therefore sought to determine whether other species with high copy number also show evidence of recent transpositional bursts. We used BLAST percent identity scores as an approximate method to estimate divergence among individual copies within species (Table 1; Figure 4). This method accurately recapitulates previous analyses for *D. yakuba *and *D. melanogaster *that were based on estimates of per-site divergence [23]: *DINE-1*s from *D. melanogaster *have a broad peak of identities centered approximately around 90%, while *D. yakuba *shows a peak from approximately 96-100%, with a long tail of more diverged copies. These differences are highly significant (Mann-Whitney *U *test, two-tailed, *p *< 0.001).

*D. simulans *and *D. sechellia *show distributions similar to *D. melanogaster *(Figure 4; *p *> 0.05) and have average percent identities around 90-91% (Table 1). *D. erecta *has an average percent identity more similar to *D. melanogaster *than to *D. yakuba*; however, its distribution is significantly different from both species (*p *< 0.001). These data suggest that *D. yakuba *is the only *melanogaster *subgroup species that experienced a recent transpositional burst. *D. grimshawii *also has a distribution with very few copies of high similarity, and a similar copy number to *D. melanogaster*, suggesting that *DINE-1 *has not been recently active in this species. In contrast, *DINE-1*s from *D. pseudoobscura*, *D. persimilis*, and *D. ananassae *have average percent identities > 95% with distributions highly skewed toward young copies, suggesting recent transpositional bursts in these species. The distributions in *D. pseudoobscura *and *D. persimilis *are significantly different (*p *< 0.001; see Discussion). *D. ananassae *in particular stands out for having many copies identical to the consensus sequence (in the block A region). *D. virilis *and *D. mojavensis *also have substantial numbers of young copies but more broad distributions, suggesting the possibility that multiple rounds of transposition, silencing and reactivation may have occurred in these species.

*D. willistoni *has more than 1,000 copies of each of its three subtypes (Table 1), with subtype 1 and 3 having about twice as many copies as subtype 2. Each subtype has a peak near 100% identity, suggesting recent transpositional activity; however, their distributions are significantly different from each other (*p *< 0.001). Interestingly, these subtypes also have different phylogenetic patterns (see below).

### Phylogenetic relationship of *DINE-1*s

These very different estimates of *DINE-1 *divergence within different species, and in particular our evidence for recent transpositional bursts, raises the question of whether *DINE-1 *may have undergone horizontal transmission into some *Drosophila *species. To understand the evolutionary dynamics of *DINE-1*s and their association with their host species, we analyzed the phylogenetic relationship of *DINE-1 *consensus sequences from the 12 *Drosophila *species (Additional data file 1) and compared it with the known phylogeny of *Drosophila *[29]. Because of the rapid evolution in the central repeat region, reliable alignment for phylogenetic reconstruction could be obtained only for blocks A and B.

**Figure 5:**
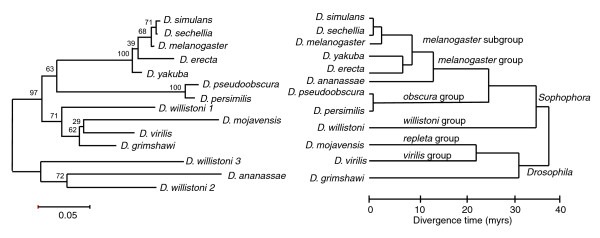
Phylogenetic relationship of *DINE-1 *consensus sequences compared to their host species. **(a) **Phylogenetic tree based on pooled sequences of block A and B (Additional data file 1) and constructed using the neighbor-joining method with the Jukes-Cantor one parameter substitution model [55]. Bootstrap resampling percentages based on 500 replications are indicated. Scale bar represents the estimated number of substitutions. **(b) **The host species phylogeny is adapted from [29]. Myrs, million years.

With the exceptions of *D. willistoni *and *D. ananassae*, the phylogenetic relationships of the *DINE-1 *sequences are, in general, consistent with the host species phylogeny (Figure 5). The grouping of *DINE-1 *in a separate clade containing *D. ananassae *and two of the three types from *D. willistoni *is surprising. The fact that this clade is an outgroup suggests that this result is not due to horizontal transfer from other *Drosophila *into these species. One possibility is that *DINE-1 *was horizontally transferred from non-*Drosophila *into these species. Alternatively, *DINE-1 *sequences resembling ancestral copies may have become reactivated in *D. ananassae *and *D. willistoni*. These sequences would be related to ancestral copies that were vertically inherited in the common ancestor of *Drosophila*.

### *DINE-1 *insertions in or near to genes

*In situ *hybridizations to polytene chromosomes from several species using species-specific *DINE-1 *probes revealed strong signals in the heterochromatic chromocenter region, with additional hybridization observed along chromosome arms (data not shown). These non-chromocenter sites of hybridization led us ask whether *DINE-1 *insertions (whole or partial) are found in or near to protein-coding genes. *SGM*-related sequences were previously found in introns or adjacent to a number of *D. melanogaster *genes [26]. We found hundreds of *DINE-1 *insertions in predicted introns in all species (Table 2; Additional data file 1). We also found many *DINE-1 *copies within 1 kb of genes, which could potentially be in either untranslated regions or in regulatory regions. These results suggest that *DINE-1 *has had a significant impact on gene structure evolution throughout the Drosophilidae. Few *DINE-1 *insertions were found in predicted coding sequences (CDSs). Strikingly, the largest number (15) was found in *D. ananassae*, a species that has a very high copy number of highly similar (young) *DINE-1 *copies (Table 1; Figure 4). This result suggests that recent transpositional activity of *DINE-1 *in *D. ananassae *has resulted in mildly deleterious insertions into coding regions that have not yet been removed by selection.

**Table 2:** Numbers of *DINE-1*s within or near to predicted genes

Species	CDS	Intron	Flanking*
*D. melanogaster*	0	669	283
*D. simulans*	0	537	282
*D. sechellia*	0	582	291
*D. yakuba*	0	717	287
*D. erecta*	0	580	280
*D. ananassae*	15	610	276
*D. persimilis*	2	460	284
*D. pseudoobscura*	0	352	277
*D. virilis*	0	1,104	278
*D. grimshawi*	0	262	174
*D. mojavensis*	2	625	292

Only block A and B sequences were used for BLAST search. *1 kb upstream and downstream of the predicted coding regions.

### Analysis of *Helitron *sequences in *D. yakuba *and *D. virilis*

Kapitonov and Jurka [24,28] recently proposed that *DINE-1 *is related to *Helitron*, a family of DNA-mediated TEs. They reported consensus sequences of autonomous and non-autonomous copies of *Helitron *in *D. yakuba *and *D. virilis*. The non-autonomous consensus sequences are closely similar to our consensus sequences reported here. The consensus autonomous copies have an open reading frame (ORF) encoding the RepHel protein found in many other *Helitrons*, and sequences at each end similar to what we report here for *DINE-1*. These include block A at the 5' end and block B at the 3' end. We searched these two species using the *RepHel *portion of the autonomous consensus sequence as a query to determine whether these species contain potentially active copies. Among the top ten hits in *D. yakuba*, none have a fully intact *RepHel *ORF. Three copies have *DINE-1 *sequences flanking both sides of the *RepHel *sequences; two of these have over 500 bp of *DINE-1 *sequence at each end while the third has only approximately 50-60 bp of *DINE-1 *sequence at each end. Six of the remaining copies have *DINE-1 *sequences flanking one side of the *RepHel *sequence, and the last hit has no flanking *DINE-1 *sequences. Among the top ten hits in *D. virilis *we again found no copies with a fully intact *RepHel *ORF. One copy has *DINE-1 *sequences flanking both sides and five copies have *DINE-1 *sequences flanking one side. Among the remaining copies, one is in a highly repetitive region and could not be further analyzed, and the remaining four copies have no flanking *DINE-1 *sequences. We conclude that *D. yakuba *and *D. virilis *are unlikely to contain currently active autonomous *Helitrons*.

## Discussion

*DINE-1 *is the most abundant repetitive sequence in the *Drosophila *genome. *DINE-1 *was first identified on the fourth chromosome of *D. melanogaster *[21], and was suggested to be a non-autonomous retroelement, analogous to vertebrate SINEs. This argument was based on its high abundance, composing > 1% of the total genome, its small size and its lack of significant ORFs [19,21,22,27,30]. However, unlike known SINEs, *D. melanogaster DINE-1 *did not appear to have polymerase III promoter consensus sequences or similarity to tRNAs or other small RNAs.

Subsequently, *DINE-1*-related sequences were found in other Dipteran species and were classified as novel TE families. Miller *et al*. [26], following earlier observations by Vivas *et al*. [25], identified *SGM *from several *obscura *group species as well as related sequences in GenBank from at least eight other *Drosophila *species, and noted its possible similarity to MITEs [26]. They further suggested that *SGM *elements composed approximately 10% of the *D. guanche *genome. Wilder and Hollocher [27] identified '*mini-me*' and characterized its sequence structure based on approximately 80 clones isolated from 2 species of the *cardini *group, *D. dunni *and *D. nigrodunni*, and 28 sequences from 14 different species obtained from GenBank. *mini-me *was classified as a non-autonomous retroelement, although no direct relationship to previously known retroelements was observed.

Previously, Yang *et al*. [23] identified a recent transpositional burst of *DINE-1 *in the genome of *D. yakuba*. The analysis of highly similar, newly inserted *DINE-1*s in this species allowed for a more detailed characterization of *DINE-1 *sequence structure. We concluded that *DINE-1 *is more likely to be a non-autonomous DNA transposon, similar to MITEs first described in maize [13], rather than a SINE-like retroelement, based on the existence of perfect terminal and subterminal inverted repeats and a TSD (TT), which are typical characteristics of DNA transposons. Moreover, the lack of polymerase III binding sites or tRNA-related structures in these recently inserted copies argued against *DINE-1 *being similar to SINEs [23]. Bergman *et al*. [6] also characterized *DINE-1 *as being a TIR transposon.

In order to understand the origin and distribution of *DINE-1 *in the Drosophilidae, we expanded our search to ten additional partial or complete *Drosophila *genome databases using the consensus sequence of *D. yakuba DINE-1*. Strikingly, we found that sequences related to *D. yakuba DINE-1 *are very abundant in all these genomes (Table 1). BLAST searches did not find any related sequences in the mosquito, silk worm or other eukaryotic genomes, suggesting that *DINE-1 *is unique to Diptera. *DINE-1*-related sequences from all the *Drosophila *species share the same sequence structure that was defined from *D. yakuba DINE-1 *or from *mini-me*, with each containing: highly conserved blocks A and B at both ends, including a core region of approximately 90 bp in block A; a central repeat region of variable length; inverted repeats 13 nucleotides long at or near the 5' end and close to the 3' end; and insertion preference for T-rich regions (Figure 2). The sequences of the central repeat region from different species are very different, suggesting non-homologous origins of this region among species. In contrast, the within species divergence of this region is much smaller.

Our comparison of *DINE-1 *from 12 species revealed a previously unobserved 3' inverted repeat structure that could potentially form a stem-loop (Figure 2). It is important to note that in the absence of any internal ORFs, the designation of 5' and 3' for *DINE-1 *is arbitrary. The presence of potential stem-loops near both ends of *DINE-1 *raises the possibility that these structures are recognized by a reverse transcriptase, which would imply that *DINE-1 *is in fact a non-autonomous retroelement. However, considering all the evidence outlined above, we suggest that *DINE-1 *transposition is DNA mediated.

It was thought previously that MITE-like DNA transposons are rare in *Drosophila*, with only a few having been identified. One example of a *Drosophila *MITE is derived from *pogo*-like transposons in *D. melanogaster *[31]. Other examples are *Vege *and *Mar*, derived from the autonomous TE *hobo *of the *hAT *superfamily in *D. willistoni *[32]. However, unlike most MITEs, which are usually highly abundant in the host genome, only a few copies (< 10) of *Vege *and *Mar *were found in the genomes of their *Drosophila *hosts [32].

Non-autonomous DNA transposons require an external source of transposase for transposition. For many TEs transposase initiates transposition by recognizing and binding to the TIR sequence, and this interaction is highly specific [33,34] Recently, Feschotte *et al*. [35] have shown that autonomous *mariner*-like transposase can not only interact with its own TIR, but can also interact with the TIR of *Stowaway *MITEs in rice. This provides strong evidence that *Stowaway *MITEs may use *mariner*-like TEs as their source for transposase.

Casola *et al*. [36] recently identified several *Drosophila *PIF-like transposons (DPLTs), which are found among *Drosophila *in both apparently autonomous and non-autonomous forms. Neither the TIR nor TSD sequences of these transposons match that of *DINE-1*, which suggests that they are not the autonomous parental copies of *DINE-1*. Intriguingly, however, *DPLT1 *has apparently active copies in *D. yakuba*, *D. pseudoobscura*, *D. persimilis *and *D. willistoni *and only inactive MITE-like copies in *D. melanogaster*, *D. simulans*, *D. sechellia*, *D. erecta *and *D. mojavensis*. This pattern closely resembles the division seen here for species that either do or do not show evidence for recent transpositional bursts of *DINE-1 *(Figure 4). These shared patterns suggest that species such as *D. yakuba *have experienced recent and ongoing movement of several DNA transposon families.

### *DINE-1*: MITE or *Helitron*?

*DINE-1 *has many features characteristic of MITEs - small size, lack of coding potential, high copy number, and frequent association with genes. On the other hand, most MITEs have TIRs, which are presumably sites of transposase binding. A few MITE-like elements have been discovered that have subTIRs rather than TIRs but their corresponding autonomous elements have not been identified [37-39]. *DINE-1 *has inverted repeats and their conservation in structure despite ongoing changes in primary sequence argues strongly that they are of functional importance. We have placed the 5' inverted repeat 0-2 nucleotides internal to the end of *DINE-1 *in different species. Under this annotation, *D. yakuba DINE-1 *insertions would not cause a TSD (Figure 3). If the true 5' end of *DINE-1 *instead corresponds to the 5' inverted repeat, then *D. yakuba DINE-1 *insertion would cause a 2 bp TSD, as seen in other MITEs. The 3' inverted repeat, however, is clearly subterminal, which would be unusual for a MITE element.

Kapitonov and Jurka [24,28] have recently proposed that *DINE-1 *is instead a non-autonomous *Helitron *element. They noted that *DINE-1 *has a number of features unusual for *Helitrons*. One was the absence of a short hairpin or palindrome at the 3' end, which is thought to function as a replication terminator. We have identified here a 3' hairpin structure in all 12 species that may fulfill this function. A number of unusual features remain. Foremost are the termini. *Helitrons *do not contain TIRs but instead have highly conserved 5' TC or 3' CTRR sequences. In contrast, *DINE-1 *lacks these short termini sequences but instead contains conserved subTIRs. The presence of relatively long blocks of conserved sequence between non-autonomous *DINE-1 *and the proposed autonomous copies also contrasts with other species. For example, bats contain several families of very high copy number non-autonomous *Helitrons*, which differ almost entirely from their autonomous master copies other than at their di- and tetra-nucleotide termini [40].

The most decisive evidence favoring the *Helitron *hypothesis is the association of *DINE-1 *elements with non-functional but recognizable partial ORFs of the RepHel protein in *D. yakuba *and *D. virilis*, making these copies the candidate autonomous elements responsible for the recent transpositional bursts of non-autonomous *DINE-1*s in these species. Considering some of the unusual features mentioned above, it will be of great interest to investigate experimentally the mechanism of *DINE-1 *transposition.

### *DINE-1 *in the *melanogaster *subgroup

From our previous study [23], we found that *D. melanogaster *and *D. yakuba *contain structurally similar types of *DINE-1*s. The species differed significantly, however, in their distributions of sequence divergence and the chromosomal location of their *DINE-1 *copies. *D. yakuba *contains many similar copies, and these apparently younger copies have a higher relative frequency in euchromatic regions compared to older, more diverged copies. We hypothesized that *DINE-1*s in *D. melanogaster *and *D. yakuba *derive from a common ancestor that existed before the divergence of the *melanogaster *subgroup species. This hypothesis was tested here by our identification of *DINE-1 *from three other species of the *melanogaster *subgroup, *D. simulans*, *D. sechellia *and *D. erecta*. All five subgroup species share the same TIRs, core, central repeat unit, and 3' end stem-loop sequences. *DINE-1*s from the three newly characterized species have similar copy numbers and distributions of sequence divergence, an observation consistent with the hypothesis that *DINE-1 *was active and then silenced in the common ancestor of the *melanogaster *subgroup. *D. yakuba *is the only species showing evidence of a second, recent transpositional burst. We did find that *DINE-1*s from *D. erecta *have a different sequence in the region joining the central repeat to block B, suggesting that this is the most rapidly evolving region of *DINE-1*.

### Dynamic nature and genomic impact of *DINE-1*

Our analysis reveals that several species outside the *melanogaster *subgroup have distributions of *DINE-1 *identity similar to that described above for *D. yakuba*, suggesting that *DINE-1 *has undergone multiple, independent transpositional bursts. *D. ananassae *and *D. willistoni *show the strongest evidence, with distributions skewed toward 100% (Figure 4). *D. virilis *has a somewhat broader distribution, with many similar copies suggestive of recent transpositional activity. *D. mojavensis *shows a broad distribution that is suggestive of multiple rounds of transposition and silencing at different times.

*D. pseudoobscura *and *D. persimilis *have distributions with peaks around 98-99% identity. These species diverged less than one million years ago [41], which might suggest that the similarly high identity in both species reflects activity of *DINE-1 *before or during their speciation. However, the distributions are significantly different, with *D. pseudoobscura *retaining proportionally more copies of high divergence. One possible explanation is that *DINE-1 *remained active more recently in *D. persimilis*. Alternatively, the strength of selection against older copies may differ between the species.

The discovery of multiple and relatively distant species each showing evidence for recently active *DINE-1 *copies raises the question of whether this element has been transmitted vertically or horizontally. The phylogenetic relationship among different *DINE-1*s (Figure 5) suggests vertical inheritance, with transpositional bursts resulting from existing copies escaping from host suppression. *DINE-1 *from *D. ananassae *and two subtypes from *D. willistoni *give a pattern discordant from the accepted species phylogeny but this pattern is also not consistent with a simple model of horizontal transfer among *Drosophila *species. Instead, we suggest that the phylogenetic pattern is likely to reflect reactivation of a related ancestral element in both *D. ananassae *and *D. willistoni*. Our analysis is necessarily limited by the relatively short sequences available for analysis. We suggest that further phylogenetic analysis of the autonomous elements from each species will help to further understand the evolution of *DINE-1*. Nevertheless, the combination of our phylogenetic analysis and the divergence data indicates that the activity of *DINE-1 *is extremely dynamic. The activation and suppression of the element seems to have evolved rapidly and repeatedly in multiple lineages leading to the 12 species.

Similar dynamics of transposition and suppression are found in LINEs and SINEs of mammalian genomes [42-44] and MITEs in plant genomes (see review in [14]). Some insertions of MITEs in plants have been shown to affect gene regulation [45,46]. We have found that *DINE-1 *insertions are frequently found in the flanking regions and introns of genes, suggesting that some copies may also influence gene regulation (Table 2).

Highly abundant interspersed repetitive sequences can also serve as targets for ectopic recombination. Such recombination may be deleterious by promoting genome instability [47,48], but may also catalyze structural evolution of existing genes and contribute to new gene formation [49]. *DINE-1 *is a candidate for causing analogous phenomenon in *Drosophila*. The testis-expressed gene *hydra *is one well-characterized example [50]. *hydra *exists only in the *melanogaster *subgroup, and its exon 1 has undergone multiple independent duplications. Many of these duplicated exon 1s are flanked by *DINE-1 *insertions, which suggests that *DINE-1 *may have facilitated some of these duplications by providing homologous target sequences for unequal crossing over. Given its high abundance and evidence for multiple rounds of transpositional activity, *DINE-1 *has clearly had a significant impact on *Drosophila *genome evolution, and we suggest that other examples of gene structural evolution associated with *DINE-1 *will be found among these species.

*DINE-1 *can also be a valuable system for studying rates and patterns of mutations. One can study *de novo *mutations in species that have had recent transpositional bursts by comparing the sequence variation among young, recently inserted *DINE-1 *copies. One can also use *DINE-1 *to examine substitution patterns between species. Previous comparative analysis of the chromosome distribution of *DINE-1 *in *D. yakuba *and *D. melanogaster *suggests that most new insertions are eliminated from the genome by negative selection [23]. Old copies that remain are thus likely to be evolving neutrally. One could therefore identify orthologous insertions between *D. melanogaster *and *D. simulans*, whose insertions must predate the divergence of these species, in order to infer the substitution pattern along lineages leading to both species. The ability to perform similar studies in multiple *Drosophila *species will allow unprecedented power for determining whether and how patterns of mutations vary in different lineages.

## Materials and methods

### Identifying *DINE-1*-related sequences from 12 *Drosophila *genomes

Using the D. yakuba DINE-1 consensus sequence as a query, we searched for DINE-1-related sequences in all 12 Drosophila genome databases (from Comparative Assembly Freeze 1 (CAF1) [51]) using BLAST with the default setting of the parameters (Figure 1). Note that D. persimilis and D. sechellia were sequenced at only approximately three- to four-fold coverage and, thus, are incomplete. We retrieved the 50 copies of DINE-1 with the lowest E-value in each species, aligned them, and derived a consensus sequence for each species (Additional data file 1).

### Sequence divergence and copy number among *DINE-1*s

We then BLAST-searched each genome using part of the consensus sequences of *DINE-1 *(5' end to end of core sequence) from its own species, using the default settings of the program. All BLAST hits greater than 100 bp were retained. The frequencies of percent identity between the query sequence and all hits were plotted for each species.

### Sequence alignment and phylogenetic analysis

Sequences were aligned using ClustalW [52] with the default parameter settings. Alignments were further improved by manual adjustment. Inferred phylogenetic trees of the species consensus of *DINE-1 *were constructed using the neighbor-joining method with bootstrap resampling (500 replicates) using MEGA 3.0 [53].

### Searching for *DINE-1 *within or near genes

The UCSC Genome Browser Gateway [54] was used to obtain locations of *DINE-1 *in the annotated genomes, with the exception of *D. willistoni*. DNA sequences were retrieved using the Genes and Gene Prediction tracks (track setting: Other RefSeq) and grouped into the following categories: category 1, 1,000 bp upstream of CDS; category 2, CDS; category 3, introns; and category 4, 1,000 bp downstream of CDS. Categories 1 and 4 were then merged into a single class of flanking sequences. We then performed BLAST search to each of these three classes of sequences, using the block A regions of the *DINE-1 *consensus of each species. Only hits longer than 40 bp with an E-value lower than 10^-5 ^were included.

### Characterizing target site duplication of *DINE-1*

From the *D. yakuba *genome database, seven sites of *DINE-1 *insertion with sequence similarity > 97% to the *D. yakuba DINE-1 *consensus were chosen for analysis. PCR primers complementary to the 100 bp flanking sequence of each site were designed (Additional data file 2). A total of ten lines of *D. yakuba*, including nine Cy lines from a natural population (gift from Dr Peter Andolfatto at UCSD) and the strain Tai18E2, which was used for whole-genome sequencing, were checked for the presence of *DINE-1 *insertions at each site. Genomic DNA was phenol-chloroform extracted from 20-30 flies per line followed by ethanol precipitation. The program for PCR reaction was: 94°C for 5 minutes, followed by 30 cycles of 94°C (30s), 60°C (30s), and 70°C (1 minute), and extension at 70°C for 7 minutes. For lines not containing the *DINE-1 *insertion, PCR products were directly sequenced using ABI BigDye (Applied Biosystems, Foster City, CA, USA) technologies.

## Abbreviations

CDS, coding sequence; *DINE-1*, *Drosophila *interspersed element 1; LINE, long interspersed elements; MITE, miniature inverted-repeat transposable element; ORF, open reading frame; SINE, short interspersed elements; TE, transposable element; TIR, terminal inverted repeat; TSD, target site duplication.

## Authors' contributions

H-PY designed the research, performed the research and analyzed the data. H-PY and DAB wrote the paper.

## Additional data files

The following additional data are available with the online version of this paper. Additional data file 1 is the alignment of *DINE-1 *consensus sequences from the 12 *Drosophila *species. Additional data file 2 is a table listing the genome locations and sequences of primers used for the presence/absence screen of *DINE-1*s in *D. yakuba*. Additional data file 3 is a table listing *DINE-1 *insertions in or near to genes.

## Supplementary Material

Additional data file 1 Alignment of *DINE-1 *consensus sequences from the 12 *Drosophila *species. Click here for file

Additional data file 2 Genome locations and sequences of primers used for the presence/absence screen of *DINE-1*s in *D. yakuba*. Click here for file

Additional data file 3 *DINE-1 *insertions in or near to genes. Click here for file
