# Supplementary material for: Abundant and species-specific DINE-1 transposable elements in 12 Drosophila genomes
Source: Genome Biol. 2008 Feb 21;9(2):R39. doi: 10.1186/gb-2008-9-2-r39 (PMC2374699; doi:10.1186/gb-2008-9-2-r39)
Supplement: Additional data file 2 — Genome locations and sequences of primers used for the presence/absence screen of DINE-1s in D. yakuba. [file gb-2008-9-2-r39-S2.doc]

**S2.** Primers used for checking presence/absence of *DINE-1* insertions at 10 sites in *D. yakuba* genome

| Site | Sequence ID | location of *DINE-1* | Forward primer | Reverse Primer |
| --- | --- | --- | --- |
| 1 | AAEU01005564.1|:21200-22293 | CCGCATCCCAATTAGTCG | CCTGGCACAATCAACAGCTA |
| 2 | AAEU01004680.1|:17404-18397 | TGCATGAGATCCTTGTCGTG | TCAGCGAATGGAAACCAGTA |
| 3 | AAEU01001179.1|:41503-42499 | CACCCACAAAATTGTTGACTC | TGCTAAGCGTTCATTGATGG |
| 4 | AAEU01003222.1|:4165-5230 | CGTCGTTTTAGGTGGCAAGT | CAGGATGCCCATTACGTAGG |
| 5 | AAEU01001917.1|:61431-62561 | CCGCCGGACATACACTTAAA | GGGCTCAAAACGAGTACCAA |
| 6 | AAEU01007822.1|:35060-36081 | GCCTCGCTCAAACTGAAAAC | TCGATCAGTTTAGGGCATCG |
| 7 | AAEU01010475.1|:39516-40708 | CCAACGAGCTATTTGCCTTT | CATTCGCTTTCGGCTAATGT |
| 8 | AAEU01001451.1|:121731-122636 | TCCTTGATTATCGGCCTCAC | CTCGAGGATCTGGCAGGA |
| 9 | AAEU01006056.1|:272005-273053 | GCTTCAGGACCAGGGAATTA | GGCCATTGGAATCAGCATAG |
| 10 | AAEU01001703.1|:95992-96971 | AGTTTACGGCCACCACATTC | ATGAAATAAGCGCCACATCG |
